# Trends in Cancer Incidence in US Adolescents and Young Adults, 1973-2015

**DOI:** 10.1001/jamanetworkopen.2020.27738

**Published:** 2020-12-01

**Authors:** Alyssa R. Scott, Kelsey C. Stoltzfus, Leila T. Tchelebi, Daniel M. Trifiletti, Eric J. Lehrer, Pooja Rao, Archie Bleyer, Nicholas G. Zaorsky

**Affiliations:** 1 Department of Radiation Oncology, Penn State Cancer Institute, Hershey, Pennsylvania; 2 Department of Public Health Sciences, Penn State College of Medicine, Hershey, Pennsylvania; 3 Department of Radiation Oncology, Mayo Clinic, Jacksonville, Florida; 4 Department of Radiation Oncology, Icahn School of Medicine at Mount Sinai, New York, New York; 5 Division of Hematology and Oncology, Department of Pediatrics, Penn State Cancer Institute, Hershey, Pennsylvania; 6 Department of Radiation Medicine, Knight Cancer Institute, Oregon Health and Science University, Portland, Oregon; 7 McGovern Medical School, University of Texas, Houston, Texas

## Abstract

**Importance:**

Previous studies have demonstrated that adolescents and young adults (AYAs) with cancer are a distinct cancer population; however, research on long-term epidemiological trends and characteristics of cancers in AYAs is lacking.

**Objective:**

To characterize the epidemiology of cancer in AYAs aged 15 to 39 years with respect to (1) patient demographic characteristics, (2) frequencies of cancer types, and (3) cancer incidence trends over time.

**Design, Setting, and Participants:**

This retrospective, serial cross-sectional, population-based study used registry data from the Surveillance, Epidemiology, and End Results (SEER) database from January 1, 1973, to December 31, 2015 (SEER 9 and SEER 18). The study population was from geographically distinct US regions, chosen to represent the racial and ethnic heterogeneity of the country. Initial analyses were performed from January 1 to August 31, 2019.

**Main Outcomes and Measures:**

Incidence rates and descriptive epidemiological statistics for patients aged 15 to 39 years with invasive cancer.

**Results:**

A total of 497 452 AYAs diagnosed from 1973 to 2015 were included in this study, with 293 848 (59.1%) female and 397 295 (79.9%) White participants. As AYAs aged, an increase in the relative incidence of carcinomas and decrease in the relative incidence of leukemias, lymphomas, germ cell and trophoblastic neoplasms, and neoplasms of the central nervous system occurred. Among the female AYAs, 72 564 (24.7%) were diagnosed with breast carcinoma; 48 865 (16.6%), thyroid carcinoma; and 33 828 (11.5%), cervix and uterus carcinoma. Among the male AYAs, 37 597 (18.5%) were diagnosed with testicular cancer; 20 850 (10.2%), melanoma; and 19 532 (9.6%), non-Hodgkin lymphoma. The rate of cancer in AYAs increased by 29.6% from 1973 to 2015, with a mean annual percentage change (APC) per 100 000 persons of 0.537 (95% CI, 0.426-0.648; *P* < .001). Kidney carcinoma increased at the greatest rate for both male (APC, 3.572; 95% CI, 3.049-4.097; *P* < .001) and female (APC, 3.632; 95% CI, 3.105-4.162; *P* < .001) AYAs.

**Conclusions and Relevance:**

In this cross-sectional, US population-based study, cancer in AYAs was shown to have a unique epidemiological pattern and is a growing health concern, with many cancer subtypes having increased in incidence from 1973 to 2015. Continued research on AYA cancers is important to understanding and addressing the distinct health concerns of this population.

## Key Points

**Question:**

What are the epidemiological characteristics and trends of cancers in US adolescents and young adults (AYAs) from 1973 to 2015?

**Findings:**

In this serial cross-sectional, US population-based study using cancer registry data from 497 452 AYAs, the rate of cancer increased by 29.6% from 1973 to 2015, with kidney carcinoma increasing at the greatest rate. Breast carcinoma and testicular cancer were the most common cancer diagnoses for female and male AYAs, respectively.

**Meaning:**

The results of this study provide further understanding of the distinct characteristics of cancer in AYAs, including incidence trends of cancer subtypes, which is valuable to guide more targeted research and interventions specifically to AYAs.

## Teaser

This cross-sectional population-based study characterizes the epidemiology of cancer in US adolescents and young adults aged 15 to 39 years with respect to (1) patient demographic characteristics, (2) cancer type frequencies, and (3) cancer incidence trends over time.

## Introduction

The National Cancer Institute has identified adolescents and young adults (AYAs) as a patient population with cancer distinct from children and older adults.^1^ Adolescents and young adults are defined as individuals aged 15 to 39 years at cancer diagnosis.^1,2^ As of 2006, approximately 70 000 AYAs were diagnosed with cancer in the US every year, and cancer was much more common in this age group than in those aged 0 to 14 years.^1,3^ Although accidents are the overall leading cause of death in adolescents, cancer is the leading cause of disease-related death in this age group.^3,4^ Despite the significant cancer burden in the AYA population, much is still unknown about the epidemiology of AYA cancers.

Although recently there has been a dramatic increase in research focused specifically on AYA cancers, historically, research has focused on childhood and adult cancers, leaving cancer in AYAs an inadequately studied problem.^2,5^ This gap has prevented the development of diagnosis, treatment, and monitoring guidelines specific to this age group.^3^ Instead, treatment and management of AYA patients often falls somewhere between pediatric and adult medical oncology frameworks, which are not ideally suited to the complex and distinct biological, social, and economic needs of AYA patients.^2,6^ In addition, subgroups of the AYA population may be at increased risk for poor outcomes.^3,7^ All of these factors make it essential to broaden the understanding of cancer epidemiology in this age group.

Although existing research has described epidemiological characteristics at a specific time and trends for limited periods (5-20 years), there remains a lack of research into the long-term trends and characteristics of cancer in AYAs.^2,8,9,10^ Our present work expands on these studies because it contains data not presented in these previous works, including incidence rates over time, mean annual percentage change (APC) values, and incidence trends for subgroups of cancer diagnoses. The objectives of this work were to characterize the epidemiology of cancer in AYAs with respect to (1) patient demographic characteristics, (2) distribution (age subgroup and primary cancer anatomical site), and (3) incidence trends from 1973 to 2015. The results of this study may be useful in identifying the evolving epidemiological characteristics and trends in cancer incidence in AYAs, a necessary next step in understanding cancer in this distinct age group.

## Methods

### Data Availability

The data used in this cross-sectional study are provided in the Surveillance, Epidemiology, and End Results (SEER) database, which is publicly accessible (https://seer.cancer.gov/). The relevant session information from the present work and abbreviated data sets (from SEER) are available elsewhere. To access the data in this study, we provide a data availability statement in the eMethods in the Supplement. These data are freely available, and thus the study was exempted from institutional review board review per policy of the Penn State College of Medicine Human Subjects Protection Office. There were no participants in this study and thus no consent form. This report follows the Strengthening the Reporting of Observational Studies in Epidemiology (STROBE) reporting guideline.

### Data Acquisition

In this retrospective, serial cross-sectional, population-based study, patients with invasive cancer diagnosed from January 1, 1973, to December 31, 2015, were abstracted from the National Cancer Institute’s SEER database. SEER is a network of population-based tumor registries from geographically distinct regions in the US, chosen to represent the racial and ethnic heterogeneity of the country. For this study, the initial SEER 9 regions representing 9% of the US were used for 1973-1999 data, and SEER 18 regions representing 28% of the US population were used for 2000-2015 data.^11,12^ The SEER registry includes data on incidence, survival, treatment (limited and on request), sex, age at diagnosis, race, marital status, and year of diagnosis. SEER*Stat, version 8.3.5 (National Cancer Institute) was used for this analysis.^12^ Patients diagnosed only through autopsy or death certificate were excluded. All incidence rates were age adjusted to the 2000 US standard population and reported per 100 000 persons.^12^ An overview and limitations of the database and the methods are described in the eMethods in the Supplement.^13,14,15,16^ Additional analyses were conducted using SAS, version 9.4 (SAS Institute Inc) and Excel, version 16.16.10 (Microsoft Corporation). Analyses were performed from January 1 to August 31, 2019. Only patients aged 15 to 39 years at diagnosis were included in this study. The AYA age subgroups were defined as 15 to 19, 20 to 24, 25 to 29, 30 to 34, and 35 to 39 years.

### Statistical Analysis

For objective 1, patient demographic characteristics were summarized by age at diagnosis, sex, and race/ethnicity. The χ^2^ test was used to determine whether significant differences exist between male and female patients and between races/ethnicities. Demographic data from patients diagnosed from 1973 to 2015 were collected from SEER 9 regions to provide an overarching picture of the AYA landscape in the US during a 42-year window. Data limited to diagnoses from 2005 to 2015 were also collected from SEER 18 regions to provide a more contemporary description of AYA cancer demographics.

For objective 2, age at diagnosis, primary cancer site, and patient count data were obtained. Disease sites were categorized based on AYA site recode/World Health Organization 2008 definition (eTable 1 in the Supplement).^17^ The SEER AYA site recode is a classification scheme for tumors specific to those aged 15 to 39 years. Because of the large number of cancer subtypes affecting AYAs, the data presented below were restricted to the 12 cancers with the highest incidence for both female and male patients.

For objective 3, incidence rates by year of diagnosis were analyzed for a statistically significant change from 1973 to 2015 using linear regression models. The APC values were comparable across scales, allowing for comparisons between incidence rate changes in rare and common cancers.^12^ Percentage change calculations were completed using 1 year as each end point, and the weighted least squares method was used to calculate APCs.^12^ A Bonferroni correction was applied to adjust for multiple hypothesis testing (n = 78 tests); 1-sided *P* < .001 was considered significant.

## Results

### AYA Cancer Demographics

A total of 497 452 patients diagnosed with invasive cancer from 1973 to 2015 were included in the demographic analysis (Table 1), including 203 604 male (40.9%) and 293 848 female (59.1%) participants. Most patients were White (397 295 [79.9%]). The remainder were Black (50 994 [10.3%]), American Indian/Alaska Native or Asian/Pacific Islander (40 753 [8.2%]), or unknown (8410 [1.7%]). The proportion of patients diagnosed with cancer increased with increasing age at diagnosis (31 645 [6.4%] at 15-19 years of age to 197 030 [39.6%] at 35-39 years of age). More female AYAs were diagnosed with cancer compared with males in every age subgroup except in those aged 15 to 19 years (14 800 [46.8%] vs 16 845 [53.2%]). Table 1 shows a significant difference in the distribution of cancer in male (40.9%) compared with female (59.1%) AYAs (*P* < .001). In addition, there was a significant difference in the distribution of cancer among people who are White (79.9%) compared with Black (10.3%), American Indian/Alaska Native/Asian/Pacific Islander (8.2%), or unknown (1.7%; *P* < .001).

**Table 1.** AYA Cancer Demographics for 1973-2015^a^

Characteristic	Age group, No. (%) of patients					
	All (N = 497 452)	15-19 y (n = 31 645)	20-24 y (n = 51 955)	25-29 y (n = 85 061)	30-34 y (n = 131 761)	35-39 y (n = 197 030)
Sex^b^						
Male	203 604 (40.9)	16 845 (53.2)	25 756 (49.6)	37 860 (44.5)	52 355 (39.7)	70 788 (35.9)
Female	293 848 (59.1)	14 800 (46.8)	26 199 (50.4)	47 201 (55.5)	79 406 (60.3)	126 242 (64.1)
Race^b^						
White	397 295 (79.9)	25 663 (81.1)	42 662 (82.1)	69 067 (81.2)	104 892 (79.6)	155 011 (78.7)
Black	50 994 (10.3)	3083 (9.7)	4594 (8.8)	7897 (9.3)	13 666 (10.4)	21 754 (11.0)
American Indian/Alaska Native or Asian/Pacific Islander	40 753 (8.2)	2451 (7.7)	3672 (7.1)	6404 (7.5)	10 886 (8.3)	17 340 (8.8)
Unknown	8410 (1.7)	448 (1.4)	1027 (2.0)	1693 (2.0)	2317 (1.8)	2925 (1.5)

Abbreviation: AYA, adolescent and young adult.

^a^ Data are from Incidence in the Surveillance, Epidemiology, and End Results Database 18 registry research data plus Hurricane Katrina–affected Louisiana cases, November 2017 submission data (1973-2015 varying), linked to county.

^b^ *P* < .001, χ^2^ analysis.

A total of 222 175 patients diagnosed from 2005 to 2015 were included in the contemporary demographic analysis (Table 2). Patterns of patient demographics for AYAs diagnosed from 2005 to 2015 were similar to those for AYAs diagnosed from 1973 to 2015.

**Table 2.** AYA Cancer Demographics for 2005-2015^a^

Characteristic	Age group, No. (%) of patients					
	All (N = 222 175)	15-19 y (n = 14 967)	20-24 y (n = 24 530)	25-29 y (n = 38 488)	30-34 y (n = 57 609)	35-39 y (n = 86 581)
Sex						
Male	87 051 (39.2)	7944 (53.1)	12 127 (49.4)	16 714 (43.4)	21 312 (37.0)	28 954 (33.4)
Female	135 124 (60.8)	7023 (46.9)	12 403 (50.6)	21 774 (56.6)	36 297 (63.0)	57 627 (66.6)
Race						
White	173 055 (77.9)	11 882 (79.4)	19 764 (80.6)	30 507 (79.3)	44 691 (77.6)	66 211 (76.5)
Black	23 198 (10.4)	1540 (10.3)	2233 (9.1)	3706 (9.6)	6042 (10.5)	9677 (11.2)
American Indian/Alaska Native or Asian/Pacific Islander	20 987 (9.4)	1284 (8.6)	1922 (7.8)	3298 (8.6)	5537 (9.6)	8946 (10.3)
Unknown	4935 (2.2)	261 (1.7)	611 (2.5)	977 (2.5)	1339 (2.3)	1747 (2.0)

Abbreviation: AYA, adolescent and young adult.

^a^ Data are from Incidence in the Surveillance, Epidemiology, and End Results Database 18 registry research data plus Hurricane Katrina–affected Louisiana cases, November 2017 submission data (1973-2015 varying), linked to county.

### Distribution of Cancers in AYAs

#### Age Subgroups and Cancer Diagnosis

Among male patients, lymphomas were the most common diagnoses in those aged 15 to 19 years (n = 4042); germ cell and trophoblastic neoplasms, for those aged 20 to 24 years (n = 7646) and 25 to 29 years (n = 10 393); and carcinomas, for those aged 30 to 34 years (n = 14 232) and 35 to 39 years (n = 27 759) (eFigure 1 and eTable 2 in the Supplement). For female patients, carcinomas were the most common diagnosis for those aged 15 to 19 years (n = 4023), 20 to 24 years (n = 11 291), 25 to 29 years (n = 27 620), 30 to 34 years (n = 55 836), and 35 to 39 years (n = 97 930).

Carcinomas were the most frequently diagnosed type of cancer for all male (n = 54 414) and female (n = 196 700) AYAs (eFigure 1 and eTable 2 in the Supplement). The relative number of carcinoma diagnoses compared with other cancer types increased as age at diagnosis increased for both male and female patients.

#### Age Subgroups and Specific Disease Site

Table 3 shows the incidence of cancer among AYAs diagnosed from 1973 to 2015 by disease site and age subgroup. eTable 2 and eFigure 2 in the Supplement show incidence data for disease sites not included in Table 3.

**Table 3.** AYA Cancer Distribution by Sex^a^

Cancer subtype^b^	Age group, No. (%)					Total No.
	15-19 y	20-24 y	25-29 y	30-34 y	35-39 y	
**Male**						
Carcinoma of the breast	0	5 (2.8)	17 (9.6)	45 (25.4)	110 (62.1)	177
Thyroid carcinoma	522 (5.1)	1092 (10.7)	1983 (19.4)	2883 (28.3)	3721 (36.5)	10 201
Melanoma	830 (4.0)	1939 (9.3)	3769 (18.1)	5902 (28.3)	8410 (40.3)	20 850
Germ cell and trophoblastic neoplasms of the gonads	2520 (6.7)	7151 (19.0)	9988 (26.6)	9895 (26.3)	8043 (21.4)	37 597
Carcinoma of the cervix and uterus	0	0	0	0	0	0
Non-Hodgkin lymphoma	1659 (8.5)	2135 (10.9)	3262 (16.7)	5186 (26.6)	7290 (37.3)	19 532
Hodgkin lymphoma	2383 (15.9)	3440 (22.9)	3476 (23.2)	3082 (20.6)	2609 (17.4)	14 990
Carcinoma of the colon and rectum	247 (2.0)	692 (5.5)	1630 (13.0)	3396 (27.1)	6580 (52.5)	12 545
Leukemias	2605 (19.7)	2217 (16.7)	2320 (17.5)	2710 (20.5)	3396 (25.6)	13 248
Kaposi sarcoma	17 (0.1)	410 (3.4)	2145 (17.9)	4454 (37.1)	4989 (41.5)	12 015
Astrocytoma	1000 (14.3)	1051 (15.0)	1305 (18.7)	1670 (23.9)	1970 (28.2)	6996
Carcinoma of kidney	84 (1.5)	202 (3.6)	588 (10.5)	1488 (26.5)	3253 (57.9)	5615
Carcinoma of trachea, bronchus, and lung	68 (1.5)	178 (3.9)	367 (8.1)	1010 (22.2)	2928 (64.3)	4551
Other sites in lip, oral cavity, and pharynx	177 (3.9)	337 (7.4)	600 (13.2)	1131 (24.8)	2314 (50.8)	4559
Carcinoma of gonads	17 (8.9)	38 (19.9)	49 (25.7)	45 (23.6)	42 (22.0)	191
**Female**						
Carcinoma of the breast	50 (0.1)	958 (1.3)	6261 (8.6)	20 051 (27.6)	45 244 (62.4)	72 564
Thyroid carcinoma	2534 (5.4)	5962 (12.7)	9747 (20.8)	13 208 (28.2)	15 414 (32.9)	46 865
Melanoma	1215 (3.8)	3846 (11.9)	6776 (21.0)	9169 (28.4)	11 239 (34.9)	32 245
Germ cell and trophoblastic neoplasms of the gonads	763 (29.9)	619 (24.3)	537 (21.1)	403 (15.8)	229 (9.0)	2551
Carcinoma of the cervix and uterus	133 (0.4)	1391 (4.1)	5686 (16.8)	11 090 (32.8)	15 528 (45.9)	33 828
Non-Hodgkin lymphoma	879 (8.0)	1310 (11.9)	2025 (18.5)	2780 (25.4)	3969 (36.2)	10 963
Hodgkin lymphoma	2449 (17.8)	3391 (24.6)	3281 (23.8)	2689 (19.5)	1985 (14.4)	13 795
Carcinoma of the colon and rectum	258 (2.3)	627 (5.5)	1458 (12.8)	3137 (27.5)	5927 (52.0)	11 407
Leukemias	1656 (17.7)	1516 (16.2)	1642 (17.5)	2048 (21.9)	2499 (26.7)	9361
Kaposi sarcoma	2 (1.2)	10 (6.1)	35 (21.3)	56 (34.1)	61 (37.2)	164
Astrocytoma	783 (15.3)	813 (15.9)	1027 (20.1)	1153 (22.5)	1346 (26.3)	5122
Carcinoma of kidney	102 (2.5)	233 (5.8)	558 (13.8)	1134 (28.1)	2012 (49.8)	4039
Carcinoma of trachea, bronchus, and lung	95 (2.1)	203 (4.5)	420 (9.4)	982 (22.0)	2771 (62.0)	4471
Other sites in lip, oral cavity, and pharynx	220 (5.9)	403 (10.8)	647 (17.4)	984 (26.4)	1468 (39.4)	3722
Carcinoma of gonads	227 (3.0)	641 (8.4)	1189 (15.6)	2032 (26.7)	3515 (46.2)	7604

Abbreviation: AYA, adolescent and young adult.

^a^ Data are from Incidence in the Surveillance, Epidemiology, and End Results Database 18 registry research data plus Hurricane Katrina–affected Louisiana cases, November 2017 submission data (1973-2015 varying), linked to county.

^b^ Sorted by decreasing absolute incidence for males and females combined and taken from the 12 disease sites with the highest absolute incidence for males and females (1973-2015).

For male AYAs, testicular cancer (germ cell and trophoblastic neoplasms of gonads) had the highest incidence (n = 37 597), followed by melanoma (n = 20 850) and non-Hodgkin lymphoma (n = 19 532). Most male AYAs with testicular cancer were diagnosed at ages 25 to 29 years (9988 [26.6%]) or 30 to 34 years (9895 [26.3%]). For both melanoma and non-Hodgkin lymphoma, the greatest number of male patients were diagnosed at ages 35 to 39 years (8410 [40.3%] and 7290 [37.3%], respectively), with decreasing incidence at younger ages at diagnosis (Table 3).

For female AYAs, carcinoma of the breast had the highest incidence (n = 72 564), followed by thyroid carcinoma (n = 46 865) and carcinoma of the cervix and uterus (n = 33 828). Most female AYAs with carcinoma of the breast (45 244 [62.4%]), thyroid carcinoma (15 414 [32.9%]), and carcinoma of the cervix and uterus (15 528 [45.9%]) were diagnosed at ages 35 to 39 years, with decreasing incidence as age subgroup decreased (Table 2).

### AYA Cancer Incidence Trends

The Figure and Table 4 show the incidence trends by disease sites for those with the 12 highest incidences for male and female AYAs from 1973 to 2015. eTable 3 in the Supplement shows changes in incidence rates for all cancer sites from 1973 to 2015. From 1973 to 2015, the incidence of cancer in AYAs increased by 0.537 (95% CI, 0.426-0.648) per 100 000 people per year (from 57.2 to 74.2 cancer diagnoses per 100 000 AYAs per year), an overall increase of 29.6% (*P* < .001).

**Figure.**
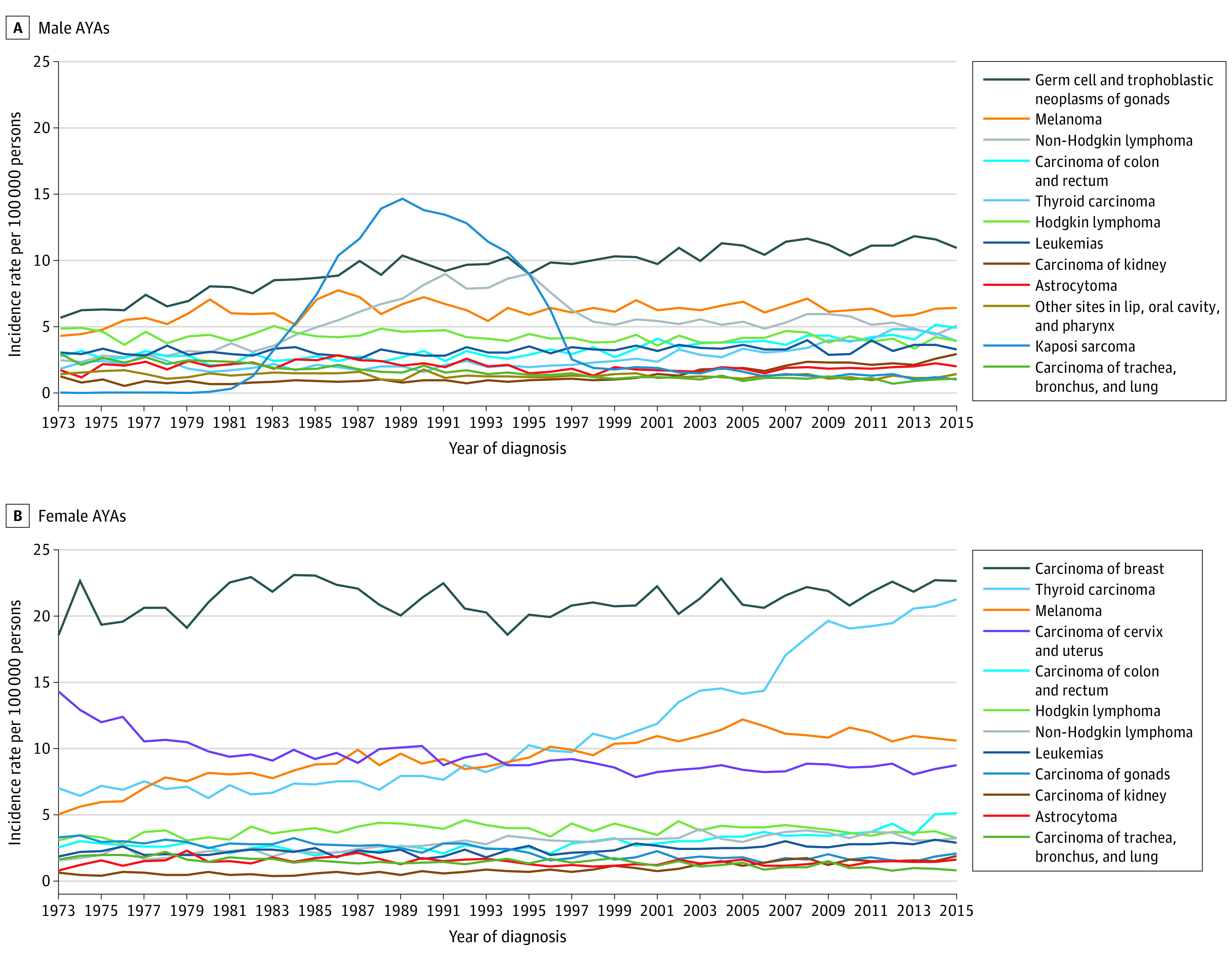
Cancer Incidence Trends for Top 12 Disease Sites With the Highest Absolute Incidence for Male and Female Adolescents and Young Adults (AYAs) From 1973 to 2015 Disease sites with the highest absolute incidences from 1973 to 2015 for both male and female AYAs were included in this figure.

**Table 4.** Trends in Cancer Incidence for Top 12 Disease Sites for Males and Females, 1973-2015^a^

Cancer subtype	Male		Female	
	APC (95% CI)	*P* value	APC (95% CI)	*P* value
Leukemias	0.406 (0.182 to 0.632)	<.001	0.864 (0.585 to 1.143)	<.001
Non-Hodgkin lymphoma	0.530 (−0.386 to 1.454)	.25	1.550 (1.224 to 1.878)	<.001
Hodgkin lymphoma	−0.323 (−0.537 to 0.109)	.004	0.191 (−0.088 to 0.471)	.17
Astrocytoma	−0.500 (−0.919 to 0.079)	.02	−0.400 (−0.858 to 0.060)	.09
Kaposi sarcoma	NA	NA	NA	NA
Germ cell and trophoblastic				
Neoplasms of gonads	1.246 (1.044 to 1.448)	<.001	0.487 (−0.027 to 1.003)	.06
Neoplasms of nongonadal sites	−0.013 (−0.636 to 0.614)	.97	−0.935 (−1.525 to −0.341)	.003
Melanoma	0.232 (−0.042 to 0.505)	.09	1.278 (1.054 to 1.501)	<.001
Thyroid carcinoma	2.273 (1.796 to 2.752)	<.001	3.456 (3.162 to 3.751)	<.001
Other sites in lip, oral cavity, and pharynx	−0.539 (−0.882 to 0.195)	.003	0.610 (0.235 to 0.987)	.002
Carcinoma				
Trachea, bronchus, and lung	−2.635 (−2.930 to −2.339)	<.001	−1.506 (−1.883 to 1.127)	<.001
Breast	NA	NA	0.107 (−0.036 to 0.249)	.14
Kidney	3.572 (3.049 to 4.097)	<.001	3.632 (3.105 to 4.162)	<.001
Cervix and uterus	NA	NA	−0.790 (−0.983 to −0.596)	<.001
Colon and rectum	1.659 (1.342 to 1.977)	<.001	1.576 (1.173 to 1.980)	<.001

Abbreviation: APC, annual percent change; NA, not available.

^a^ Data are from the Surveillance, Epidemiology, and End Results Database 9, November 2017 submission data (1973-2015) adjusted for Hurricanes Katrina and Rita and linked to county attributes.

Among male patients from 1973 to 2015, 14 disease sites had a statistically significant increase in incidence (eTable 3 in the Supplement). Disease sites with the greatest increase in incidence were carcinoma of the kidney (APC, 3.572 [95% CI, 3.049-4.097]; *P* < .001), unspecified soft tissue sarcoma (APC, 2.543 [95% CI, 1.811-3.280]; *P* < .001), and thyroid carcinoma (APC, 2.273 [95% CI, 1.796-2.752]; *P* < .001). Seven disease sites had a statistically significant decrease in incidence, with astrocytoma not otherwise specified (APC, −3.759 [95% CI, −4.534 to −2.978]; *P* < .001) and carcinoma of the trachea, bronchus, and lung (APC, −2.635 [95% CI, −2.930 to −2.339]; *P* < .001) having the greatest decreases in incidence (eTable 3 in the Supplement).

Among female patients from 1973 to 2015, 15 cancer disease sites had a statistically significant increase in incidence (eTable 3 in the Supplement). Disease sites with the greatest increase in incidence were carcinoma of the kidney (APC, 3.632 [95% CI, 3.105-4.162]; *P* < .001), thyroid carcinoma (APC, 3.456 [95% CI, 3.162-3.751]; *P* < .001), and myeloma, mast cell, and miscellaneous lymphoreticular neoplasms not otherwise specified (APC, 2.805 [95% CI, 1.923-3.694]; *P* < .001). Five disease sites had a statistically significant decrease in incidence, with astrocytoma not otherwise specified (APC, −3.369 [95% CI, −4.289 to −2.441]; *P* < .001) and carcinoma of the gonads (APC, −1.743 [95% CI, −2.064 to −1.420]; *P* < .001) having the greatest decreases in incidence (eTable 3 in the Supplement).

## Discussion

This cross-sectional study found that AYAs are affected by a wide variety of cancers, and the diagnosis of these cancers has been increasing from 1973 to 2015. During this period, the rate of cancer in AYAs increased by 29.6%, from 57.2 to 74.2 invasive cancer diagnoses per 100 000 AYAs per year. In contrast, previous findings assessed trends in cancer incidence for different age ranges.^18^ In addition, from 1975 to 1995, most pediatric cancers did not significantly increase or decrease in incidence, and from 1992 to 2004, there was no significant increase in the incidence rate for pediatric cancers.^19,20^

The 29.6% increase in the rate of cancer in AYAs reflects the increasing rates of many cancer types. Carcinoma of the kidney, thyroid, and gastrointestinal tract (eg, carcinoma of the colon and rectum), germ cell and trophoblastic neoplasms (eg, testicular cancer), and melanoma, among other cancer sites, have been increasing in AYAs during this period (eTable 3 in the Supplement). Although carcinoma of the kidney has been increasing at the greatest rate, its contribution to the overall increased incidence may be minimal owing to its relatively low absolute incidence during 1973 to 2015. Cancer sites with larger absolute incidences contribute more to the overall increase in cancers among AYAs. These sites include carcinoma of the gastrointestinal tract, thyroid carcinoma, melanoma, non-Hodgkin lymphoma, and germ cell and trophoblastic neoplasms of the gonads.

The increasing rates of diagnosis at many of the disease sites identified in this work have been observed in previous analyses of data from more historical and limited periods.^21,22,23,24,25,26^ The present study demonstrates that the rate of diagnosis of cancer at these sites has increased from 1973 through 2015.

Environmental factors, dietary and obesity trends, and changes in screening practices are 3 major categories that likely played a role in the increasing rate of cancer in AYAs from 1973 to 2015. For example, factors that may be involved in the increasing rate of testicular cancer include exposure to estrogens and progesterone (prenatally and via dairy consumption), increased prevalence of maternal risk factors (increasing age at first birth, having fewer children, and increasing survival of premature infants), and exposure to cannabis.^21,27,28,29^ Diet and obesity may play a role in the development of thyroid carcinoma and carcinoma of the kidney.^9,30,31^ In addition, cancer screening and overdiagnosis are thought to account for much of the increasing rates of thyroid and kidney carcinoma, among others.^32^ Worsening diet (less vegetables and more fats and processed meats), increasing rates of childhood and adolescent obesity, lack of exercise, and human papillomavirus infections have been implicated as potential risk factors contributing to the increasing rate of gastrointestinal tract cancers among AYAs.^9,22,33,34,35^ The increasing rate of diagnosis of melanoma in female AYAs may be associated with increasing intensity of UV exposure, including the use of artificial UV sources.^36^ Increasing exposure to chemotherapy and ionizing radiation may be another factor contributing to the overall increase in AYA cancers.^37^

This study also found a decrease in incidence in 7 disease sites in male patients and 5 disease sites in female patients (eTable 3 in the Supplement). Potential explanations for the decreasing rates for some AYA disease sites include increased oral contraceptive use, laws restricting exposure to environmental toxins (eg, benzene), and decreased smoking initiation.^9,38,39^ Lung, breast, colorectal, bladder, and endometrial cancers are among those most influenced by lifestyle and environmental factors.^40^ As shown in the Figure, reduced sun and other UV exposure (eg, tanning salons) has been associated with melanoma, especially in females (eg, carcinoma of the trachea, bronchus, lung, and bladder) (Figure, B).

Consistent with previous literature, in this study, the incidence of Kaposi sarcoma and non-Hodgkin lymphoma in men dramatically increased in the 1980s and then decreased in the 1990s (Figure and Table 4). This pattern of incidence for these cancers has been shown to be associated with the onset and consequent decrease of the HIV/AIDS epidemic in the 1980s and 1990s.^41^

Adolescents and young adults are a heterogenous group. They are affected by a wide variety of cancers, and the distribution of the type of cancer diagnoses shifts throughout the AYA age range. As AYAs age, there is generally a decrease in the relative incidence of leukemias, lymphomas, germ cell and trophoblastic neoplasms, and neoplasms of the central nervous system and an increase in the relative incidence of carcinomas (Table 3 and eTable 2 in the Supplement). This outcome is consistent with studies that have looked at epidemiological data from 1973 to 1982, 1995 to 1999, and 2011 to 2015, which found that the incidence of nonepithelial cancers (eg, lymphoma, leukemia, testicular cancer, and neoplasms of the central nervous system) decreased compared with epithelial cancers (eg, carcinomas) as people in the AYA age range get older.^8,10,42^ Factors to consider that may be associated with this change include hormone levels during puberty and pregnancy and cancers related to environmental exposures (later onset) vs genetic factors (earlier onset).^10,42^ The present study suggests that this shift from nonepithelial to epithelial cancers within the AYA age group is characteristic of the 1973 to 2015 time period.

Demographic patterns of cancer from 1973 to 2015 are consistent with more contemporary demographic patterns from 2005 to 2015 (Tables 1 and 2). In every age subgroup, female AYAs outnumber male AYAs except those aged 15 to 19 years, in which males are diagnosed at a greater rate. This difference may be owing to the shift from nonepithelial to epithelial cancers as AYAs age: the decrease in disease sites commonly affecting male AYAs (eg, testicular cancer, leukemias, and lymphomas) coinciding with the increase in cancers that are major contributors to mortality in female AYAs (eg, carcinoma of the breast).

### Limitations

A limitation of this study is that the data from SEER is limited to people living in the US, and therefore our results may not be generalizable to other countries. In addition, data from SEER are limited to collection at the time of diagnosis and do not include information about recurrence. Finally, reporting of race/ethnicity may contain errors, resulting in underestimates of cancer in some subpopulations.

## Conclusions

Despite cancer in AYAs being a significant and growing problem, data on the epidemiology of cancer in AYAs are limited.^5,10^ Previous research has shown that AYA cancers have distinct features from cancers in other age groups, and subgroups of the AYA population may be at increased risk for poor outcomes.^7,43,44,45,46^ In this cross-sectional, US population-based study using cancer registry data from 497 452 AYAs, the rate of cancer increased by 29.6% from 1973 to 2015, with kidney carcinoma increasing at the greatest rate. Breast carcinoma and testicular cancer were the most common cancer diagnoses for female and male AYAs, respectively. This study demonstrated that cancer in AYAs has a distinct distribution and is a growing problem and that continued research in this area is necessary to address the unique needs of this population. Specifically, there is a need to use the expanded knowledge about AYA cancers to target diagnosis and treatment options specific to the unique patterns and characteristics of cancer in this age group.

## Notes

Supplement. **eMethods.** Surveillance, Epidemiology, and End Results (SEER) Database **eTable 1.** AYA Site Recode/WHO 2008 Definition **eTable 2.** Incidence of All Cancer Subtypes, 1973-2015 **eTable 3.** Trends in Cancer Incidence, 1973-2015 **eFigure 1.** Cancer Diagnosis by Age at Diagnosis and Cancer Type **eFigure 2.** Cancer Diagnoses by Disease Site and Age at Diagnosis **eReferences.**
