# Supplementary material for: Trends in Cancer Incidence in US Adolescents and Young Adults, 1973-2015
Source: JAMA Netw Open. 2020 Dec 1;3(12):e2027738. doi: 10.1001/jamanetworkopen.2020.27738 (PMC7709088; doi:10.1001/jamanetworkopen.2020.27738)
Supplement: Supplement. — eMethods. Surveillance, Epidemiology, and End Results (SEER) Database eTable 1. AYA Site Recode/WHO 2008 Definition eTable 2. Incidence of All Cancer Subtypes, 1973-2015 eTable 3. Trends in Cancer Incidence, 1973-2015 eFigure 1. Cancer Diagnosis by Age at Diagnosis and Cancer Type eFigure 2. Cancer Diagnoses by Disease Site and Age at Diagnosis eReferences. [file jamanetwopen-e2027738-s001.pdf]

Supplementary Online Content

Scott AR, Stoltzfus KC, Tchelebi LT, et al. Trends in cancer incidence in US adolescents and young adults, 1973-2015. *JAMA Netw Open*. 2020;3(12):e2027738. doi:10.1001/jamanetworkopen.2020.27738

eMethods. Surveillance, Epidemiology, and End Results (SEER) Database

eTable 1. AYA Site Recode/WHO 2008 Definition

eTable 2. Incidence of All Cancer Subtypes, 1973-2015

eTable 3. Trends in Cancer Incidence, 1973-2015

eFigure 1. Cancer Diagnosis by Age at Diagnosis and Cancer Type

eFigure 2. Cancer Diagnoses by Disease Site and Age at Diagnosis

eReferences.

This supplementary material has been provided by the authors to give readers additional information about their work.

©2020 Scott AR et al. *JAMA Network Open*.

eMethods. Surveillance, Epidemiology, and End Results (SEER) Database

Calendar period, registry, and diversity

The SEER program has evolved since its inception in the United States in 1973.[1] As of 2017, SEER has up to 36 years of longitudinal and ongoing data collection, with a representative sample size of more than 6 million cancer cases, and a comprehensive quality assurance process. Over time, more registries were added to SEER; in the current analysis, the SEER 9 and 18 (adjusted for Hurricane Katrina Impacted Louisiana cases) were used. The registry number denotes the number of registries. *SEER 9.* The first areas included at that time were Connecticut, Hawaii, Iowa, San Francisco/Oakland, and Detroit. Geographic areas were included based on two objectives: (1) the ability of a geographic cancer registry to maintain high-quality data (explained below), and (2) having a population that represents minority subpopulations.[1]

In 1974-1975, the metropolitan areas of Atlanta and Seattle/Puget Sound were added, and the SEER 9 registry was finalized. *SEER 11*. In order to expand on the second objective, two more registries were added, Los Angeles County and 4 Counties in the San Jose/Monterey area. These counties included cases diagnosed after 1992. *SEER 13*. The next grouping additionally included 10 predominantly African American counties of rural Georgia and the Alaska Native American Tumor Registry. *SEER 17*. For cancers diagnoses after 2001, four additional areas were included: the remaining counties of California, Kentucky, Louisiana, and New Jersey. These counties have supplemental funding by the Centers for Disease Control (CDC). Based on the inclusion of these areas, the SEER database is representative of the population of the USA, and this has been validated by external studies.[1]

Since the SEER database have increased the proportion of the US population captured over the years, in early years of the SEER program there are fewer survivors than in later years, and the proportion of death by index cancer is lower in later years. Further, the rate count of people having a cancer depends on the number of patients living with this cancer from previous years (which depends on cancer prevalence), those diagnosed within the calendar year (which depends on screening and incidence), and those dying during that year (which depends on cancer and treatment aggressiveness, how death is coded, common risk factors among cancers and comorbidities, and patient age). Certain cancers have an indolent course (e.g. prostate), and patients diagnosed in subsequent years are added to the cumulative count, increasing the number of prostate cancer patients relative to all others; for patients with aggressive cancers (e.g. pancreatic), the addition of patients diagnosed in subsequent years has little effect on the cumulative number because of high rates of mortality.

Quality assurance and completeness

SEER undergoes quality assurance using systematic, standardized, and periodic data collection procedure for all defined members of a defined cohort is performed to avoid surveillance bias.[1] The case-finding audits are performed by a qualified member from each SEER registry under the direction of members of the National Cancer Institute. Auditors create an abstract the contains the primary site and the case finding source.[2] When performing audits, SEER adheres to two basic principles: auditing high quantity and high-risk data. High quantity refers to disease sites that have the highest incidence and prevalence (e.g. breast, prostate, lung, colon); as well facilities that contribute the greatest percent of cases to the central database. Additionally, pathology laboratories are selected to review tissue from patients not seen at that hospital. High risk refers to cases that are likely to be miscoded (e.g. head and neck, hematopoietic diseases); compliance to new rules; and newly-reportable diseases.

Data availability statement (DAS)

The instructions to access the SEER data are provided below:

(1) Download the SEER*Stat software from the NCI website: https://seer.cancer.gov/seerstat/software/

(2) Open the program

(3) Click “File”, “New,”

“Frequency Session” to generate the number of cases. Note, this was used in Figure 1 and 2 of the current analysis.

“Case Listing” to generate a list of patient cases diagnosed. Note, this was used in Table 1 (demographic table) of the current analysis.

©2020 Scott AR et al. *JAMA Network Open*.

“Rate Session” to generate a list of the incidence rates of cancers. Note, this was used in Figure 3 and Table 2 of the current analysis.

(4) Click on the desired registry to use for each of the sessions. All the other data supporting the findings of this study are available within the article and its supplementary information files and from the corresponding author upon reasonable request

eTable 1. AYA Site Recode/WHO 2008 Definition

Site Group	ICD-O-3 Behavior Recode	Primary Site	ICD-O-3 Histology	Recode
**1.Leukemias**				
1.1 Acute lymphoid leukemia	3	C000-C809	9826, 9835-9836	01
	3	C420-C421, C424	9811-9818, 9837	01
1.2 Acute myeloid leukemia	3	C000-C809	9840, 9861, 9865-9867, 9869, 9871-9874, 9891, 9895-9898, 9910-9911, 9920	02
1.3 Chronic myeloid leukemia	3	C000-C809	9863, 9875-9876, 9945-9946	03
1.4 Other and unspecified leukemia	3	C000-C809	9742, 9800-9801, 9805-9809, 9820, 9831-9834, 9860, 9870, 9930-9931, 9940, 9948, 9963-9964	04
	3	C420-C421, C424	9823, 9827	04
**2. Lymphomas**				
2.1 Non-Hodgkin lymphoma	3	C000-C809	9590-9591, 9596-9597, 9670-9671, 9673, 9675, 9678-9680, 9684, 9687-9691, 9695, 9698-9702, 9705, 9708-9709, 9712, 9714, 9716-9719, 9725-9729, 9735, 9737-9738	05
	3	C000-C419, C422-C423, C425-C809	9811-9818, 9823, 9827, 9837	05
2.2 Hodgkin lymphoma	3	C000-C809	9650-9655, 9659, 9661-9665, 9667	06
**3. CNS and Other Intracranial and Intraspinal Neoplasms (all behaviors)**				
3.1. Astrocytoma				
3.1.1 Specified low-grade astrocytic tumors	0, 1, 3	C723	9380	07
	0, 1, 3	C000-C809	9410-9411, 9420-9421, 9424	07
3.1.2 Glioblastoma and anaplastic astrocytoma	0, 1, 3	C000-C809	9401, 9440-9442	08
3.1.3 Astrocytoma, NOS	0, 1, 3	C000-C809	9400	09
3.2 Other glioma	0, 1, 3	C000-C722, C724-C809	9380	10
	0, 1, 3	C000-C809	9381-9384, 9423, 9430, 9450-9451, 9460	10

©2020 Scott AR et al. *JAMA Network Open*.

3.3 Ependymoma	0, 1, 3	C000-C809	9391-9394	11
3.4 Medulloblastoma and other PNET				
3.4.1 Medulloblastoma	0, 1, 3	C716	9470-9474	12
3.4.2 Supratentorial PNET	0, 1, 3	C000-C715, C717-C809	9470-9474	13
3.5 Other specified intracranial and intraspinal neoplasms	0, 1, 3	C000-C699, C730-C750, C754-C809	9350-9351, 9360-9362, 9390, 9480, 9530-9535, 9537-9539, 9541, 9550, 9562, 9570	14
	0, 1, 3	C700-C729, C751-C753	9161, 9361-9362, 9390, 9530-9531, 9535, 9538, 9540, 9560, 9571	14
	0, 1, 3	C700	9532, 9534, 9537, 9539	14
	0, 1, 3	C753	9360	14
	0, 1, 3	C711	9480, 9539	14
	0, 1, 3	C713	9480, 9533	14
	0, 1, 3	C719	9350	14
	0, 1, 3	C714, C717	9480	14
	0, 1, 3	C709	9539	14
3.6 Unspecified intracranial and intraspinal neoplasms				
3.6.1 Unspecified malignant intracranial and intraspinal neoplasms	3	C700-C729, C751-C753	8000-8005	15
3.6.2 Unspec. ben/border intracran. and intraspin. Neo.	0, 1	C700-C729, C751-C753	8000-8005	16
**4. Osseous & Chondromatous Neoplasms**				
4.1 Osteosarcoma	3	C000-C809	9180-9187, 9192-9194	17
4.2 Chondrosarcoma	3	C000-C809	9220-9221, 9230-9231, 9240, 9242-9243	18
4.3 Ewing tumor	3	C000-C809	9260, 9364-9365	19
4.4 Other specified and unspecified bone tumors	3	C000-C809	8812, 9250, 9261, 9370-9372	20
	3	C400-C419	8000-8005, 8800-8803, 8805-8806, 9200	20
**5. Soft Tissue Sarcomas**				

©2020 Scott AR et al. *JAMA Network Open*.

5.1 Fibromatous neoplasms	3	C000-C809	8810-8811, 8813-8815, 8820-8824, 8830, 8832-8833, 8835-8836, 9252	21
5.2 Rhabdomyosarcoma	3	C000-C809	8900-8904, 8910, 8912, 8920-8921, 8991	22
5.3 Other soft tissue sarcoma				
5.3.1 Specified soft tissue sarcoma				
5.3.1.1 Specified (excluding Kaposi sarcoma)	3	C000-C809	8804, 8825, 8840-8897, 8982-8983, 8990, 9040-9044, 9120-9139, 9141-9150, 9170, 9251, 9561, 9580-9581	23
	3	C000-C699, C730-C750, C754-C809	9540, 9560, 9571	23
5.3.1.2 Kaposi sarcoma	3	C000-C809	9140	24
5.3.2 Unspecified soft tissue sarcoma	3	C000-C399, C420-C809	8800-8803, 8805-8806	25
**6. Germ Cell and Trophoblastic Neoplasms**				
6.1 Germ cell and trophoblastic neoplasms of gonads	3	C569, C620-C629	9060-9065, 9070-9073, 9080-9085, 9090-9091, 9100-9102, 9105	26
6.2 Germ cell and trophoblastic neoplasms of nongonadal sites				
6.2.1 Intracranial (all behaviors)	0, 1, 3	C700-C729, C751-C753	9060-9065, 9070-9073, 9080-9085, 9090-9091, 9100-9102, 9105	27
6.2.2 Other nongonadal	3	C000-C568, C570-C619, C630-C699, C730-C750, C754-C809	9060-9065, 9070-9073, 9080-9085, 9090-9091, 9100-9102, 9104-9105	28
**7. Melanoma and Skin Carcinomas**				
7.1 Melanoma	3	C000-C809	8720-8723, 8726, 8728, 8730, 8740-8746, 8761, 8770-8774, 8780	29
7.2 Skin carcinomas	3	C440-C449	8010-8589	30
**8. Carcinomas**				
8.1 Thyroid carcinoma	3	C739	8010-8589	31
8.2 Other carcinoma of head and neck				
8.2.1 Nasopharyngeal carcinoma	3	C110-C119	8010-8589	32
8.2.2 Other sites in lip, oral cavity and pharynx	3	C000-C109, C120-C148	8010-8589	33

©2020 Scott AR et al. *JAMA Network Open*.

8.2.3 Nasal cav, mid ear, sinuses, larynx, oth ill-def head/neck	3	C300-C329, C760	8010-8589	34
8.3 Carcinoma of trachea, bronchus, and lung	3	C330-C349	8010-8589	35
8.4 Carcinoma of breast	3	C500-C509	8010-8589	36
8.5 Carcinoma of genitourinary tract				
8.5.1 Carcinoma of kidney	3	C649	8010-8589	37
8.5.2 Carcinoma of bladder	3	C670-C679	8010-8589	38
8.5.3 Carcinoma of gonads	3	C569, C620-C629	8010-8589	39
	3	C000-C809	8590-8593	39
8.5.4 Carcinoma of cervix and uterus	3	C530-C559	8010-8589	40
8.5.5 Carc of other and ill-def sites, geniourinary tract	3	C510-C529, C570-C579, C600-C619, C630-C639, C659, C669, C680-C689	8010-8589	41
8.6 Carcinoma of gastrointestinal tract				
8.6.1 Carcinoma of colon and rectum	3	C180-C218	8010-8589	42
8.6.2 Carcinoma of stomach	3	C160-C169	8010-8589	43
8.6.3 Carcinoma of liver and intrahepatic bile ducts	3	C220-C221	8010-8589	44
8.6.4 Carcinoma of pancreas	3	C250-C259	8010-8589	45
8.6.5 Carc other and ill-def sites, gastrointestinal tract	3	C150-C159, C170-C179, C230-C249, C260-C269	8010-8589	46
8.7 Carcinoma of other and ill-def sites				
8.7.1 Adrenocortical carcinoma	3	C740-C749	8010-8589	47
8.7.2 Carcinoma of other and ill-defined sites, NOS	3	C149, C219, C222-C229, C270-C299, C350-C439, C450-C499, C561-C568, C580-C599, C640-C648, C650-C658, C660-C668, C690-C738, C750-C759, C761-C809	8010-8589	48
	3	C809	9010	48
**9. Miscellaneous specified neoplasms, NOS**				
9.1 Other pediatric and embryonal tumors, NOS				
9.1.1 Wilms tumor	3	C000-C809	8959-8960	49

©2020 Scott AR et al. *JAMA Network Open*.

9.1.2 Neuroblastoma	3	C000-C809	9490, 9500	50
9.1.3 Other pediatric and embryonal tumors, NOS	3	C000-C809	8963-8964, 8970-8973, 8981, 9363, 9501-9523	51
9.2 Other specified and embryonal tumors, NOS				
9.2.1 Paraganglioma and glomus tumors	3	C000-C809	8680-8711	52
9.2.2 Other specified gonadal tumors	3	C000-C809	8600-8650, 9000	53
	3	C569	8670, 9013-9015, 9054	53
9.2.3 Myeloma, mast cell, misc. lymphoreticular neo., NOS	3	C000-C809	9724, 9731-9734, 9740-9741, 9743-9764, 9766, 9769, 9960, 9965-9967, 9970-9971	54
9.2.4 Other specified neoplasms, NOS	3	C000-C809	8930-8951, 8980, 9020, 9050-9053, 9110, 9160, 9270-9330, 9950, 9961-9962, 9975, 9980, 9982, 9989, 9991-9992	55
	3	C000-C699, C730-C750, C754-C809	9161	55
**10. Unspecified Malignant Neoplasms**	3	C000-C399, C420-C699, C730-C750, C754-C809	8000-8005	56
**Unclassified**				99

(source: https://seer.cancer.gov/ayarecode/aya-who2008.html)

©2020 Scott AR et al. *JAMA Network Open*.

eTable 2. Incidence of All Cancer Subtypes, 1973-2015

Cancer Subtypes	*Males*						*Females*					
	15-19	***20-24***	***25-29***	***30-34***	***35-39***	***Total***	***15-19***	***20-24***	***25-29***	***30-34***	***35-39***	***Total***
1 Leukemias	2,605	2,217	2,320	2,710	3,396	13,248	1,656	1,516	1,642	2,048	2,499	9,361
1.1 Acute lymphoid leukemia	1,582	876	626	553	551	4,188	767	418	371	379	365	2,300
1.2 Acute myeloid leukemia	701	801	882	1,020	1,151	4,555	651	754	825	981	1,136	4,347
1.3 Chronic myeloid leukemia	183	366	602	742	932	2,825	146	239	307	466	565	1,723
1.4 Other and unspecified leukemia	139	174	210	395	762	1,680	92	105	139	222	433	991
2 Lymphomas	4,042	5,575	6,738	8,268	9,899	34,522	3,328	4,701	5,306	5,469	5,954	24,758
2.1 Non-Hodgkin lymphoma	1,659	2,135	3,262	5,186	7,290	19,532	879	1,310	2,025	2,780	3,969	10,963
2.2 Hodgkin lymphoma	2,383	3,440	3,476	3,082	2,609	14,990	2,449	3,391	3,281	2,689	1,985	13,795
3 CNS and Oth Intracranial and Intraspinal Neo (all behav)	1,718	1,847	2,401	2,931	3,314	12,211	1,374	1,483	1,911	2,126	2,413	9,307
3.1. Astrocytoma	1,000	1,051	1,305	1,670	1,970	6,996	783	813	1,027	1,153	1,346	5,122
3.1.1 Specified low-grade astrocytic tumors	458	292	267	310	248	1,575	389	275	224	229	209	1,326
3.1.2 Glioblastoma and anaplastic astrocytoma	269	396	589	841	1,182	3,277	165	285	452	524	741	2,167
3.1.3 Astrocytoma, NOS	273	363	449	519	540	2,144	229	253	351	400	396	1,629
3.2 Other glioma	316	405	686	875	940	3,222	279	368	532	648	712	2,539
3.3 Ependymoma	115	112	146	151	180	704	92	111	137	142	155	637
3.4. Medulloblastoma and other PNET	226	207	183	127	103	846	157	128	123	103	85	596
3.4.1 Medulloblastoma	129	132	105	88	63	517	87	76	74	69	59	365
3.4.2 Supratentorial PNET	97	75	78	39	40	329	70	52	49	34	26	231
3.5 Other specified intracranial and intraspinal neoplasms	35	42	38	47	63	225	44	40	48	46	70	248
3.6 Unspecified intracranial and intraspinal neoplasms	26	30	43	61	58	218	19	23	44	34	45	165
3.6.1 Unspec malignant intracranial and intraspinal neo	26	30	43	61	58	218	19	23	44	34	45	165
3.6.2 Unspec ben/border intracran. and intraspin neo	0	0	0	0	0	0	0	0	0	0	0	0
4 Osseous & Chondromatous Neoplasms	1,571	833	624	558	585	4,171	839	588	505	416	463	2,811
4.1 Osteosarcoma	836	343	175	138	143	1,635	442	249	181	123	122	1,117
4.2 Chondrosarcoma	111	132	183	222	224	872	59	103	138	156	171	627
4.3 Ewing tumor	554	285	173	113	87	1,212	284	163	111	62	82	702
4.4 Other specified and unspecified bone tumors	70	73	93	85	131	452	54	73	75	75	88	365
5 Soft Tissue Sarcomas	1,140	1,770	3,828	6,537	7,406	20,681	1,020	1,280	1,646	2,124	2,698	8,768
5.1 Fibromatous neoplasms	250	389	615	787	886	2,927	290	472	682	832	855	3,131
5.2 Rhabdomyosarcoma	314	164	90	74	78	720	209	85	65	61	43	463
5.3 Other soft tissue sarcoma	576	1,217	3,123	5,676	6,442	17,034	521	723	899	1,231	1,800	5,174
5.3.1 Specified soft tissue sarcoma	422	997	2,891	5,411	6,153	15,874	411	562	736	1,042	1,520	4,271
5.3.1.1 Specified (excluding Kaposi sarcoma)	405	587	746	957	1,164	3,859	409	552	701	986	1,459	4,107
5.3.1.2 Kaposi sarcoma	17	410	2,145	4,454	4,989	12,015	2	10	35	56	61	164
5.3.2 Unspecified soft tissue sarcoma	154	220	232	265	289	1,160	110	161	163	189	280	903
6 Germ Cell and Trophoblastic Neoplasms	3,029	7,646	10,393	10,221	8,273	39,562	965	911	874	705	476	3,931
6.1 Germ cell and trophoblastic neoplasms of gonads	2,520	7,151	9,988	9,895	8,043	37,597	763	619	537	403	229	2,551
6.2 Germ cell and trophoblastic neo of nongonadal sites	509	495	405	326	230	1,965	202	292	337	302	247	1,380
6.2.1 Intracranial (all behaviors)	323	167	88	49	14	641	55	17	11	11	9	103

©2020 Scott AR et al. *JAMA Network Open*.

6.2.2 Other nongonadal	186	328	317	277	216	1,324	147	275	326	291	238	1,277
7 Melanoma and Skin Carcinomas	843	1,958	3,807	6,005	8,545	21,158	1,229	3,875	6,826	9,233	11,357	32,520
7.1 Melanoma	830	1,939	3,769	5,902	8,410	20,850	1,215	3,846	6,776	9,169	11,239	32,245
7.2 Skin carcinomas	13	19	38	103	135	308	14	29	50	64	118	275
8 Carcinomas	1,642	3,561	7,220	14,232	27,759	54,414	4,023	11,291	27,620	55,836	97,930	196,700
8.1 Thyroid carcinoma	522	1,092	1,983	2,883	3,721	10,201	2,534	5,962	9,747	13,208	15,414	46,865
8.2 Other carcinoma of head and neck	344	529	862	1,697	3,440	6,872	305	518	833	1,284	2,023	4,963
8.2.1 Nasopharyngeal carcinoma	137	140	154	315	482	1,228	70	73	100	153	239	635
8.2.2 Other sites in lip, oral cavity and pharynx	177	337	600	1,131	2,314	4,559	220	403	647	984	1,468	3,722
8.2.3 Nasal cav,mid ear,sinus,larynx,ill-def head/neck	30	52	108	251	644	1,085	15	42	86	147	316	606
8.3 Carcinoma of trachea,bronchus, and lung	68	178	367	1,010	2,928	4,551	95	203	420	982	2,771	4,471
8.4 Carcinoma of breast	0	5	17	45	110	177	50	958	6,261	20,051	45,244	72,564
8.5 Carcinoma of genitourinary tract	222	523	1,187	2,739	5,940	10,611	536	2,448	7,845	15,064	22,669	48,562
8.5.1 Carcinoma of kidney	84	202	588	1,488	3,253	5,615	102	233	558	1,134	2,012	4,039
8.5.2 Carcinoma of bladder	110	253	470	986	1,871	3,690	51	113	228	357	696	1,445
8.5.3 Carcinoma of gonads	17	38	49	45	42	191	227	641	1,189	2,032	3,515	7,604
8.5.4 Carcinoma of cervix and uterus	0	0	0	0	0	0	133	1,391	5,686	11,090	15,528	33,828
8.5.5 Carc of oth and ill-def sites, geniourinary tract	11	30	80	220	774	1,115	23	70	184	451	918	1,646
8.6 Carcinoma of gastrointestinal tract	417	1,061	2,470	5,255	10,580	19,783	425	991	2,169	4,612	8,755	16,952
8.6.1 Carcinoma of colon and rectum	247	692	1,630	3,396	6,580	12,545	258	627	1,458	3,137	5,927	11,407
8.6.2 Carcinoma of stomach	29	107	298	707	1,290	2,431	37	112	282	645	1,119	2,195
8.6.3 Carcinoma of liver and intrahepatic bile ducts	86	137	246	403	801	1,673	73	104	137	203	384	901
8.6.4 Carcinoma of pancreas	27	51	102	322	880	1,382	49	86	154	338	673	1,300
8.6.5 Carc oth and ill-def sites, gastrointestinal tract	28	74	194	427	1,029	1,752	8	62	138	289	652	1,149
8.7 Carcinoma of other and ill-def sites	69	173	334	603	1,040	2,219	78	211	345	635	1,054	2,323
8.7.1 Adrenocortical carcinoma	12	21	27	47	66	173	22	33	37	59	87	238
8.7.2 Carcinoma of other and ill-defined sites, NOS	57	152	307	556	974	2,046	56	178	308	576	967	2,085
9 Miscellaneous specified neoplasms, NOS	211	230	343	654	1,178	2,616	312	363	592	966	1,697	3,930
9.1 Other pediatric and embryonal tumors, NOS	109	67	58	66	65	365	96	69	54	61	69	349
9.1.1 Wilms tumor	18	15	7	9	8	57	29	20	13	9	10	81
9.1.2 Neuroblastoma	44	24	14	15	9	106	36	23	18	16	13	106
9.1.3 Other pediatric and embryonal tumors, NOS	47	28	37	42	48	202	31	26	23	36	46	162
9.2 Other specified and embryonal tumors, NOS	102	163	285	588	1,113	2,251	216	294	538	905	1,628	3,581
9.2.1 Paraganglioma and glomus tumors	19	18	38	54	49	178	18	26	21	35	39	139
9.2.2 Other specified gonadal tumors	7	18	19	24	31	99	70	56	101	135	191	553
9.2.3 Myeloma, mast cell, misc lymphoreticular neo, NOS	45	75	124	333	733	1,310	31	49	85	209	488	862
9.2.4 Other specified neoplasms, NOS	31	52	104	177	300	664	97	163	331	526	910	2,027
10 Unspecified Malignant Neoplasms	44	119	185	239	433	1020	54	190	279	481	755	1,759
Unclassified and Non-Malignant	0	0	1	0	0	1	0	1	0	2	0	3

©2020 Scott AR et al. *JAMA Network Open*.

eTable 3. Trends in Cancer Incidence, 1973-2015

Cancer Subtype	*Males*		*Females*	
	***APC**	***P-Value***	***APC**	***P-Value***
1 Leukemias	0.406	0.0007	**0.864**	**<0.0001**
1.1 Acute lymphoid leukemia	0.994	0.0002	**2.045**	**<0.0001**
1.2 Acute myeloid leukemia	0.328	0.067	**0.771**	**<0.0001**
1.3 Chronic myeloid leukemia	0.374	0.081	0.274	0.30
1.4 Other and unspecified leukemia	-0.359	0.18	-0.169	0.63
2 Lymphomas	0.278	0.27	**0.773**	**<0.0001**
2.1 Non-Hodgkin lymphoma	0.530	0.25	**1.550**	**<0.0001**
2.2 Hodgkin lymphoma	-0.323	0.004	0.191	0.17
3 CNS and Oth Intracranial and Intraspinal Neo (all behav)	0.356	0.0046	0.549	0.0003
3.1. Astrocytoma	-0.500	0.021	-0.400	0.087
3.1.1 Specified low-grade astrocytic tumors	**2.012**	**<0.0001**	-	-
3.1.2 Glioblastoma and anaplastic astrocytoma	0.730	0.006	0.417	0.15
3.1.3 Astrocytoma, NOS	**-3.759**	**<0.0001**	**-3.369**	**<0.0001**
3.2 Other glioma	**2.041**	**<0.0001**	**2.241**	**<0.0001**
3.3 Ependymoma	1.315	0.0013	2.049	0.0011
3.4. Medulloblastoma and other PNET	0.934	0.092	1.806	0.0038
3.4.1 Medulloblastoma	-0.386	0.45	-	-
3.4.2 Supratentorial PNET	-	-	-	-
3.5 Other specified intracranial and intraspinal neoplasms	-	-	-	-
3.6 Unspecified intracranial and intraspinal neoplasms	-	-	-	-
3.6.1 Unspec malignant intracranial and intraspinal neo	-	-	-	-
3.6.2 Unspec ben/border intracran. and intraspin neo	-	-	-	-
4 Osseous & Chondromatous Neoplasms	0.690	0.0001	**1.160**	**<0.0001**
4.1 Osteosarcoma	0.373	0.24	0.634	0.041
4.2 Chondrosarcoma	0.552	0.20	0.829	0.12
4.3 Ewing tumor	1.367	0.0001	**2.312**	**<0.0001**
4.4 Other specified and unspecified bone tumors	0.108	0.86	-	-
5 Soft Tissue Sarcomas	-2.744	0.013	-0.062	0.66
5.1 Fibromatous neoplasms	-0.525	0.055	-0.432	0.095
5.2 Rhabdomyosarcoma	-0.503	0.25	0.340	0.46
5.3 Other soft tissue sarcoma	-3.889	0.0028	0.099	0.60
5.3.1 Specified soft tissue sarcoma	-4.439	0.0015	-0.252	0.22
5.3.1.1 Specified (excluding Kaposi sarcoma)	**0.905**	**<0.0001**	-0.289	0.16
5.3.1.2 Kaposi sarcoma	-	-	-	-
5.3.2 Unspecified soft tissue sarcoma	**2.543**	**<0.0001**	**2.092**	**<0.0001**
6 Germ Cell and Trophoblastic Neoplasms	**1.181**	**<0.0001**	-0.022	0.91
6.1 Germ cell and trophoblastic neoplasms of gonads	**1.246**	**<0.0001**	0.487	0.063
6.2 Germ cell and trophoblastic neo of nongonadal sites	-0.013	0.97	-0.935	0.0028
6.2.1 Intracranial (all behaviors)	0.000	-	0.000	-
6.2.2 Other nongonadal	-0.802	0.031	-1.075	0.0012
7 Melanoma and Skin Carcinomas	0.244	0.077	**1.282**	**<0.0001**

©2020 Scott AR et al. *JAMA Network Open*.

7.1 Melanoma	0.232	0.094	**1.278**	**<0.0001**
7.2 Skin carcinomas	-	-	-	-
8 Carcinomas	**0.658**	**<0.0001**	**0.676**	**<0.0001**
8.1 Thyroid carcinoma	**2.273**	**<0.0001**	**3.456**	**<0.0001**
8.2 Other carcinoma of head and neck	**-0.619**	**<0.0001**	0.429	0.011
8.2.1 Nasopharyngeal carcinoma	-0.047	0.88	0.626	0.11
8.2.2 Other sites in lip, oral cavity and pharynx	-0.539	0.003	0.610	0.0021
8.2.3 Nasal cav,mid ear,sinus,larynx,ill-def head/neck	**-1.521**	**<0.0001**	-1.029	0.028
8.3 Carcinoma of trachea,bronchus, and lung	**-2.635**	**<0.0001**	**-1.506**	**<0.0001**
8.4 Carcinoma of breast	-	-	0.107	0.14
8.5 Carcinoma of genitourinary tract	**1.001**	**<0.0001**	**-0.679**	**<0.0001**
8.5.1 Carcinoma of kidney	**3.572**	**<0.0001**	**3.632**	**<0.0001**
8.5.2 Carcinoma of bladder	**-1.706**	**<0.0001**	-1.403	0.0002
8.5.3 Carcinoma of gonads	-	-	**-1.743**	**<0.0001**
8.5.4 Carcinoma of cervix and uterus	-	-	**-0.790**	**<0.0001**
8.5.5 Carc of oth and ill-def sites, geniourinary tract	1.229	0.0117	-0.854	0.003
8.6 Carcinoma of gastrointestinal tract	**1.423**	**<0.0001**	**1.535**	**<0.0001**
8.6.1 Carcinoma of colon and rectum	**1.659**	**<0.0001**	**1.576**	**<0.0001**
8.6.2 Carcinoma of stomach	-0.123	0.66	1.086	0.0001
8.6.3 Carcinoma of liver and intrahepatic bile ducts	**2.113**	**<0.0001**	0.942	0.027
8.6.4 Carcinoma of pancreas	0.331	0.35	**1.828**	**<0.0001**
8.6.5 Carc oth and ill-def sites, gastrointestinal tract	**2.039**	**<0.0001**	**1.911**	**<0.0001**
8.7 Carcinoma of other and ill-def sites	**-1.817**	**<0.0001**	-0.571	0.041
8.7.1 Adrenocortical carcinoma	0.000	-	0.064	0.046
8.7.2 Carcinoma of other and ill-defined sites, NOS	**-1.926**	**<0.0001**	-0.696	0.017
9 Miscellaneous specified neoplasms, NOS	**1.439**	**<0.0001**	**1.396**	**<0.0001**
9.1 Other pediatric and embryonal tumors, NOS	0.137	0.82	-0.531	0.26
9.1.1 Wilms tumor	-	-	-	-
9.1.2 Neuroblastoma	-	-	-	-
9.1.3 Other pediatric and embryonal tumors, NOS	-	-	-	-
9.2 Other specified and embryonal tumors, NOS	**1.632**	**<0.0001**	**1.646**	**<0.0001**
9.2.1 Paraganglioma and glomus tumors	-	-	-	-
9.2.2 Other specified gonadal tumors	-	-	1.711	0.0093
9.2.3 Myeloma, mast cell, misc lymphoreticular neo, NOS	**1.490**	**<0.0001**	**2.805**	**<0.0001**
9.2.4 Other specified neoplasms, NOS	1.283	0.025	1.185	0.0001
10 Unspecified Malignant Neoplasms	-0.154	0.69	0.233	0.54
Unclassified and Non-Malignant	-	-	-	-

Bold= significant (p<0.0001) change in incidence
*APC= annual percent change

©2020 Scott AR et al. *JAMA Network Open*.

eFigure 1. Cancer Diagnosis by Age at Diagnosis and Cancer Type

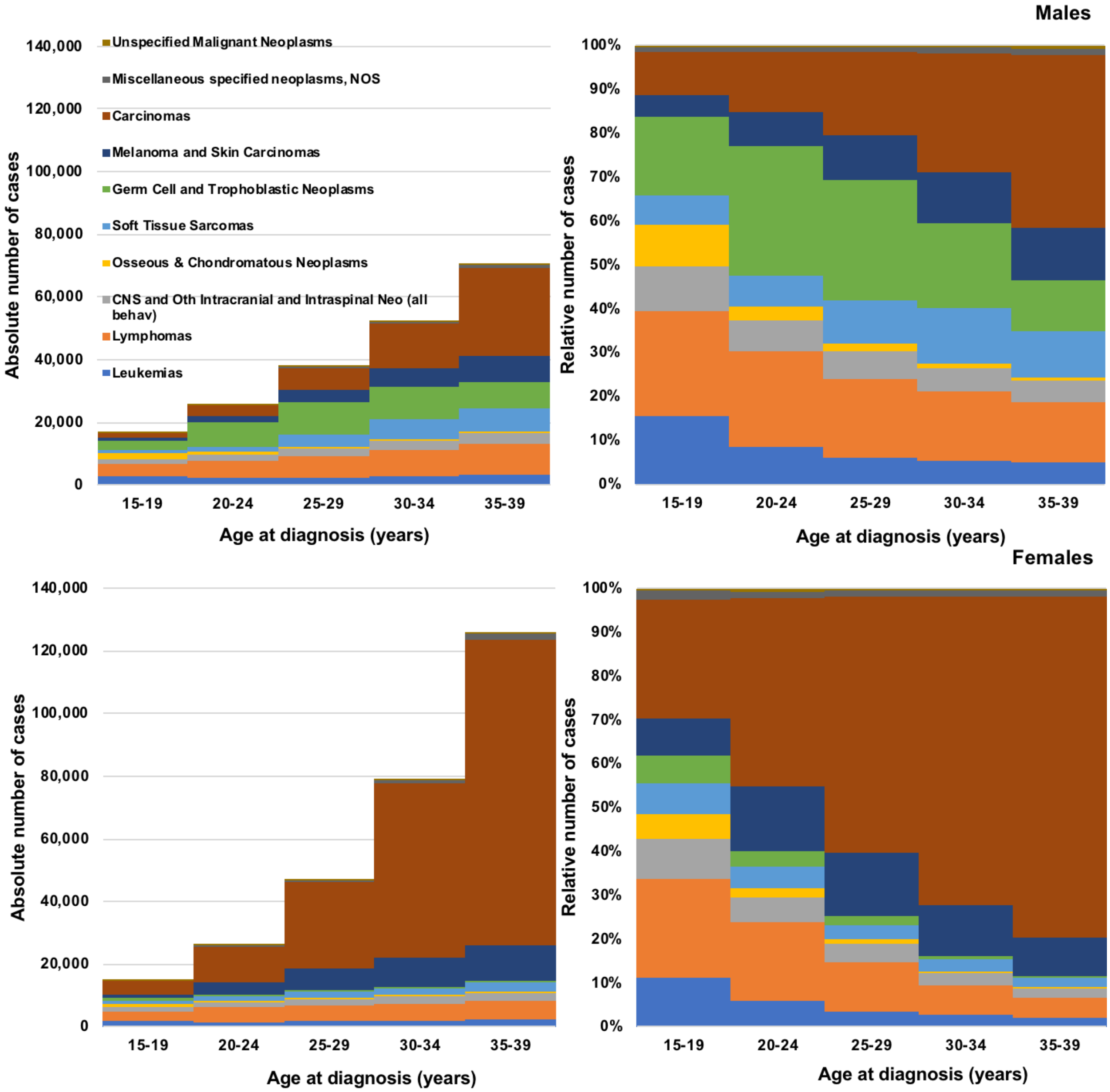

Supplementary figure 1. Adolescent and Young Adult (AYA) cancer diagnoses by age at diagnosis and cancer type.

The y-axes depict the absolute and relative number of cancer diagnoses from 1973 to 2015. The x-axes depict the age subgroup at diagnosis. Different colors represent cancer type classified based on AYA site recode/World Health Organization 2008 definition.

©2020 Scott AR et al. *JAMA Network Open*.

eFigure 2. Cancer Diagnoses by Disease Site and Age at Diagnosis

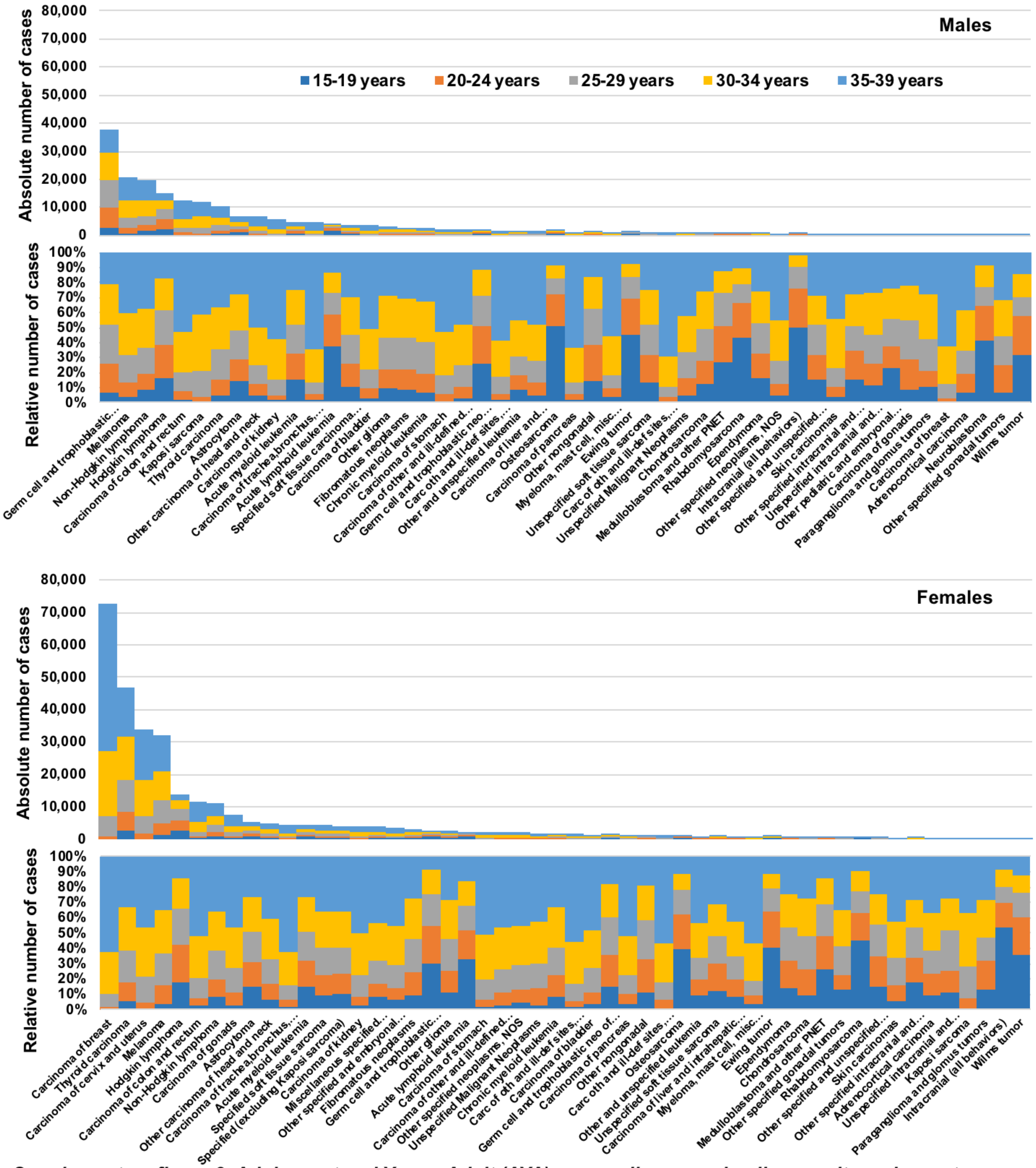

Supplementary figure 2. Adolescent and Young Adult (AYA) cancer diagnoses by disease site and age at diagnosis.

The y-axes depict the absolute and relative number of cancer diagnoses from 1973 to 2015. The x-axes depict the disease site (based on AYA site recode/World Health Organization 2008 definition), and different colors represent the age subgroup at diagnosis.

©2020 Scott AR et al. *JAMA Network Open*.

eReferences

1. Park, H., et al., *Overview of the Surveillance, Epidemiology, and End Results Database: Evolution, Data Variables, and Quality Assurance.* Vol. 36. 2012. 183-90*.*
2. National Cancer Institute. *Casefinding Studies-SEER Quality Improvement*. 2016; Available from: http://seer.cancer.gov/qi/tools/casefinding.html.

©2020 Scott AR et al. *JAMA Network Open*.
